# Direct-Write Spray Coating of a Full-Duplex Antenna for E-Textile Applications

**DOI:** 10.3390/mi11121056

**Published:** 2020-11-29

**Authors:** Ying Zhou, Saber Soltani, Braden M. Li, Yuhao Wu, Inhwan Kim, Henry Soewardiman, Douglas H. Werner, Jesse S. Jur

**Affiliations:** 1 Department of Textile Engineering, Chemistry and Science, North Carolina State University, Raleigh, NC 27606, USA; yzhou32@ncsu.edu (Y.Z.); bmli@ncsu.edu (B.M.L.); ikim7@ncsu.edu (I.K.); 2 Electrical Engineering Department, The Pennsylvania State University, University Park, PA 16802, USA; saber_soltani@psu.edu (S.S.); yuw476@psu.edu (Y.W.); dhw@psu.edu (D.H.W.); 3 Mechanical and Aerospace Engineering, North Carolina State University, Raleigh, NC 27606, USA; hsoewar@ncsu.edu

**Keywords:** flexible textile antenna, screen printing, direct-write spray coating, microstrip patch antenna

## Abstract

Recent advancements in printing technologies have greatly improved the fabrication efficiency of flexible and wearable electronics. Electronic textiles (E-textiles) garner particular interest because of their innate and desirable properties (i.e., conformability, breathability, fabric hand), which make them the ideal platform for creating wireless body area networks (WBANs) for wearable healthcare applications. However, current WBANs are limited in use due to a lack of flexible antennas that can provide effective wireless communication and data transfer. In this work, we detail a novel fabrication process for flexible textile-based multifunctional antennas with enhanced dielectric properties. Our fabrication process relies on direct-write printing of a dielectric ink consisting of ultraviolet (UV)-curable acrylates and urethane as well as 4 wt.% 200 nm barium titanate (BT) nanoparticles to enhance the dielectric properties of the naturally porous textile architecture. By controlling the spray-coating process parameters of BT dielectric ink on knit fabrics, the dielectric constant is enhanced from 1.43 to 1.61, while preserving the flexibility and air permeability of the fabric. The novel combination textile substrate shows great flexibility, as only 2 N is required for a 30 mm deformation. The final textile antenna is multifunctional in the sense that it is capable of operating in a full-duplex mode while presenting a relatively high gain of 9.12 dB at 2.3 GHz and a bandwidth of 79 MHz (2.260–2.339 GHz) for each port. Our proposed manufacturing process shows the potential to simplify the assembly of traditionally complex E-textile systems.

## 1. Introduction

In the past decade, wireless body area networks (WBANs) have garnered significant attention as a way to wirelessly communicate between wearable devices placed at various locations across the human body [1,2,3]. These wearable devices can sense physiological signals, such as electrocardiogram (ECG), electroencephalogram (EEG), and electrooculogram (EOG) [4]. Moreover, these devices, when coupled with WBANs, create wearable health monitoring systems that enable a paradigm shift away from traditional, in-person healthcare towards remote telemedicine. A primary challenge to WBANs is that they primarily rely on conventional stiff and heavy antenna designs to wirelessly relay information between devices, which limits practical applications of WBANs in wearables. Having a wearable antenna that is flexible and lightweight, with the same efficiency as a conventional, rigid antenna, is critical for realizing WBANs for telemedicine applications [5,6]. It is also important that there is a pathway to fabricating multifunctional wearable antennas, which typically require more complex geometrical features.

The antenna is a necessary wireless communication device for receiving and transmitting relevant signals at specific frequencies [7]. Various wearable antennas with flexible properties exist in the literature, using a range of polymer- and paper-based substrates with permittivity values often ranging from 1.5 to 13 [8,9]. Textile materials, made of fibrous polymer materials, are ubiquitous in our daily lives, and they offer a light and flexible substrate for wearable systems and devices. The textile antennas [10,11,12] can be light, flexible, and readily integrated into smart clothing, communicating with other wearable electronics and transmitting the body health data [1]. Design criteria, such as durability, functionality, and usability, need to be met in order to design convenient and safe textile antennas [13]. The safety impact of a textile antenna is defined by its specific absorption rate (SAR) [14], which measures the rate of absorption of radio-frequency (RF) electromagnetic radiation per unit mass by a human body to evaluate the RF dosimetry [15]. Ideally, all wearable antennas should exhibit low SARs to ensure a low negative impact on human tissues [16,17].

With these design criteria in mind, researchers have explored multiple textile designs, materials, and manufacturing methods to realize textile-based antennas. For example, researchers have used conductive yarns embroidered into non-conductive woven fabrics to create wearable patch antennas [18]. These embroidered textile antennas are easy assemble and integrate into wearable systems. However, embroidery can be challenging for large-scale manufacturing processes. In addition, these embroidered antennas show low patch resolution and low wash durability, as the conductive yarns are susceptible to degradation during washing and handling. Nonwoven textiles serve as an alternate material substrate for textile-based antennas [19]. However, these types of antennas employ copper tape as the patch and ground, which make them very fragile, and they are easily broken after a few bending cycles [20]. Printing methods, such as screen printing, are currently the best processes to design textile-based antennas with excellent wash and mechanical durability [20,21,22]. In our previous work, we reported the use of a nonwoven fabric assembly laminated with a thermoplastic polyurethane (TPU) web to create a porous, flexible, and washable antenna [19]. Employing the breathable TPU web in the screen-printed antenna can work as a protection layer by mechanically entrapping the dielectric materials to enhance their durability [19]. Moreover, the micro-fiber TPU web with an open gap structure enabled breathability. The TPU web encapsulated silver printed on textiles, protecting the conductive area of the antenna from being washed and mechanically deformed [19,21]. A recent textile antenna made of screen-printed nonwoven fabrics laminated with a TPU web was reported, and a novel dual-port design was used to improve the isolation with enhanced bandwidths [23]. The antenna showed a low profile and an SAR < 0.37 W/kg [23]. However, there is still room for improvement regarding the flexibility of these antennas. Current literature focuses on the metallic layers in textile-based antennas rather than the substrate itself. The essential dielectric layers in textile-based antennas play a big role in the antennas’ performance. Since the inherent porous structure of textiles limits the ability to achieve a high dielectric constant, it is necessary to reduce the surface wave losses and improve the antenna’s bandwidth [24]. A higher substrate dielectric constant is also needed for the downsizing of antennas [7]. Although knit fabrics constructed with yarn loops and large air voids are widely used in daily clothing due to their flexibility and comfort, they typically have very low dielectric constants due to the highly porous structure [25]. This characteristic limits their application as substrates for wearable antennas.

In this work, a multifunctional full-duplex textile-based antenna is introduced with dual ports and a highly flexible nonwoven and knit composite antenna substrate. The antenna is fabricated with a screen-printed patch and ground on a nonwoven fabric and sandwiched onto knit fabrics with a spray-coated dielectric layer. Spray coating is a high-throughput direct-write method that can drive film deposition on a printed substrate to change its dielectric properties. It has been widely used due to its high speed (up to 200 mm/s). Additionally, inks with a wide range of viscosities from 0.7 to 2500 mPa s can be printed [26,27]. To overcome the low dielectric constant of the knit fabric, a dielectric-particle-loaded ink was applied to the fabric by spray coating. The measurement results show that the radiation performance of the fabricated antenna is robust to structural deformation and agrees with the simulated results.

By employing a new fabrication process and material system, a much more flexible antenna substrate can be achieved while, at the same time, its equivalent relative permittivity is increased. As a consequence, for the same design area, the effective wavelength is larger compared to our previously published work [23]. The fabricated antenna exhibits a 79 MHz bandwidth at a center frequency of 2.3 GHz with a remarkable 30 dB isolation between ports, which represents a 15 dB improvement in isolation compared to [23], and a relatively high gain of 9.12 dB compared with other textile antennas [22,28,29,30,31]. 

## 2. Materials and Methods

### 2.1. Preparation and Characterization of the Barium Titanate Ultraviolet (UV) Curing Ink

A dielectric ink for direct-write spray coating was prepared by mixing 4 wt.% BaTiO_3_ (BT) nanoparticles (200 nm, purchased from Nanostructured Amorphous Materials Inc., Houston, TX, USA) with a urethane and acrylate UV-curable dielectric ink (C3-D1–1032 Dielectric photopolymer, Chem3, LLC, Stony Brook, NY, USA). The mixture was then stirred at 400 rpm for 24 h in order to disperse the nanoparticles, followed by an ultrasonic homogenizing process for 30 min. The solid BT content in the ink was fixed at 4 wt.% in the entire experiment.

The viscosity of the ink under different shear rates was tested using a rheometer (MCR 302, Anton Paar). Surface tension of the ink was tested by a contact angle goniometer (First Ten Angstroms Europe; Cambridge, UK) with an automated syringe to form the pendant drop. The nanoparticle crystal structure phase was tested using a Rigaku SmartLab X-ray Diffractometer (XRD) with a CuKα X-ray radiation source (λ = 0.154 nm). The XRD pattern was recorded from 2-theta = 10° to 60° at a scanning rate of 3°/min with a step size of 0.05°.

A 3D laser-scanning confocal microscope (Keyence, model VK-X1000) and a field emission electron microscope (FESEM) model FEI Quanta 650 were used to characterize the morphology of fabrics printed with conductive ink and BT dielectric ink.

### 2.2. Configuration of Antenna

**Figure 1:**
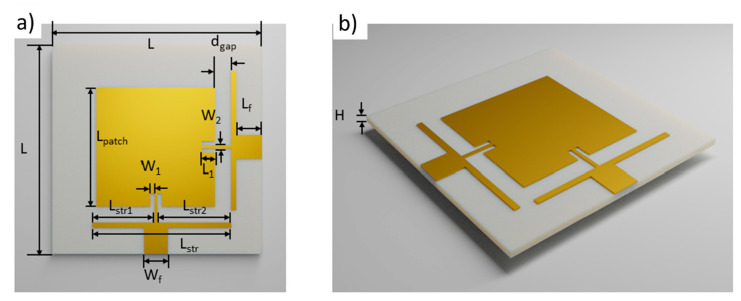
Geometrical parameters of the dual-port full-duplex textile-based patch antenna. (**a**) Top view. (**b**) Side view. Design dimensions: H = 2 mm, L = 80 mm, L_patch_ = 45.8 mm, L_str1_ = 19.1 mm, L_str2_ = 25.5 mm, L_str_ = 53.6 mm, L_f_ = 6 mm, W_f_ = 7 mm, L_1_ = 4.5 mm, W_1_ = 1 mm, W_2_ = 0.8 mm, and d_gap_ = 7 mm [23].

The flexible antenna design had two separated ports with a high isolation between them to enable transmitting (Tx) and receiving (Rx) functions (i.e., full-duplex operation). Figure 1 shows the configuration of the proposed wearable antenna, which is similar to the design in our previously published work [23]. The thickness of the textile substrate was 2 mm, which was much thinner than other wearable antennas published in the literature [31,32,33,34,35]. In comparison to a large majority of patch antenna designs, our antenna design used a strip line placed perpendicularly across the two feeds to enhance the bandwidth and isolation between the two ports. The active and ground conductive layers show thicknesses of around 40 μm and conductivity of 1.3 × 10^6^ s/m. The dielectric constant of the antenna is 1.78 and dielectric loss is 0.021. Based on this information, the optimized design parameters listed in Figure 1 were determined using an ANSYS High-Frequency Structural Simulator (HFSS). In Figure 1, when Port-1 is excited, the electromagnetic energy couples to the strip near Port-2. This feature not only significantly suppresses the mutual coupling between the two feed ports, but also improves impedance matching of the patch antenna and broadens the bandwidth [23]. The same coupling effect can also be observed on the other strip load when Port-2 is excited, but with the orthogonally aligned surface current distribution. The unidirectional radiation patterns were also similar when either Port-1 or Port-2 was excited. The antenna design has a high isolation of more than 30 dB via the strip load coupling.

### 2.3. Dielectric Constant Test

Traditional techniques for permittivity measurements at microwave frequencies can be divided into non-resonant methods and resonant methods [36]. Non-resonant methods mainly use transmission/reflection measurements, but have limited resolution. For our analysis, we used the split post dielectric resonator (SPDR) method to test the permittivity of the samples without damaging them. A 2.45 GHz SPDR was connected to a two-port vector network analyzer (VNA) by high-precision coaxial cables to analyze the samples [37]. The device resonates at a specific frequency with a quality factor (Q-factor) over 2000. Because of the high Q factor, it is more sensitive to the dielectric samples than a conventional coil-and-capacitor resonant circuit [38]. The sample size requirement for the SPDR test is at least 5 cm × 5 cm, and the sample thickness should be less than 2 mm. The dielectric constant measurements were repeated five times. This method can provide rapid and accurate results at high frequency.

### 2.4. Antenna Fabrication

Nonwoven Evolon^®®^ fabric (30 wt.% polyamide and 70 wt.% polyester) was obtained from Freudenberg Performance Materials. This nonwoven fabric is an excellent choice for electronics printing due to the numerous condensed fibers exhibiting a high surface area and low surface roughness compared to conventional textiles. Single jersey-knit fabrics composed of 100% polyester (PET) with a basis weight of 145 g/m^2^ were obtained from Hanesbrands Inc. Both the nonwoven and knit fabric thicknesses were ~0.3 mm, as shown in Table 1. Prior work has demonstrated that stacking the nonwoven layers can result in a good substrate for antenna fabrication [23]. The commercial knit structure is generally too flexible to keep the antenna stable. Therefore, a combination structure with alternating layers of printed knit fabrics and nonwoven fabrics was chosen for the antenna fabrication. The structured device was assembled with six layers of printed knit fabrics adhered together by a porous TPU web (0.057 mm). The layered knit fabric and TPU structure were heat laminated at 150 °C for 5 min. The TPU web softens above 65 °C and melts under higher temperatures.

An Asymtek C-341 conformal coating machine fitted with an EFD-781 s series spray valve was used to print the BT dielectric ink on the knit textile to modify the dielectric constant of the substrate. The BT dielectric ink was loaded into a syringe and capped with a plunger. The dispensing pressure was 20 psi, and the air-assist pressure was set to 60 psi. These settings enabled uniform ejection of ink from the spray valve. The processing included a dispensing velocity of 40 mm/s and a gap of 1 mm between the nozzle and substrate.

**Figure 2:**
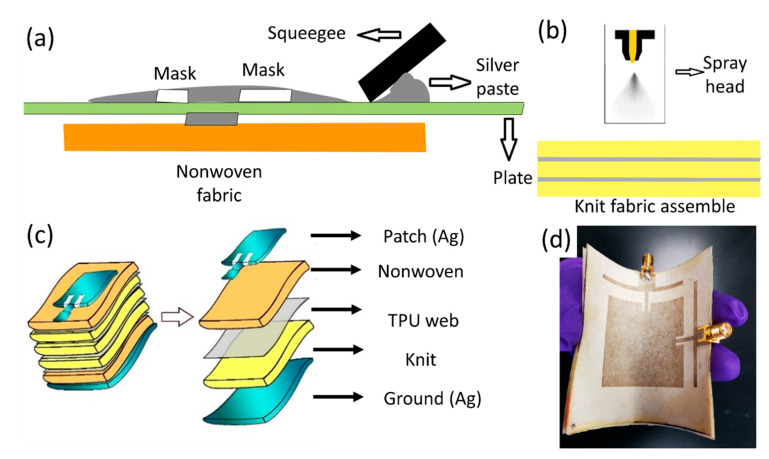
Fabrication of the textile-based antenna. (**a**) Screen printing of the Ag paste from Dupont onto nonwoven fabrics to fabricate the patch and ground. (**b**) Direct-write spray coating of barium titanate (BT) UV curable dielectric ink onto the knit fabric substrate assembly (six layers of knit fabric laminated with a thermoplastic polyurethane (TPU) web) to modify the dielectric constant. (**c**) Final assembly of the printed textile-based antenna (two layers of nonwoven fabric and six layers of knit fabric spray coated with BT dielectric ink). Exploded views indicate the different layer compositions. (**d**) Final product image of the printed textile-based antenna.

Figure 2 shows the assembly process of the textile-based antenna. The first step in the fabrication process was to heat press six knit fabrics adhered to TPU webs. Then, the BT dielectric ink was spray coated onto the knit fabric composite to enhance its dielectric constant and was matched to the nonwoven fabrics (Figure 2b). Finally, the fabric coated with the BT dielectric ink was cured under a UV radiation machine for 300 s to make sure the photopolymer was completely cured.

The patch and ground patterns were screen printed using a DuPont 5064 H conductive screen-printing silver ink on individual nonwoven fabrics (Figure 2a). The nonwoven Evolon^®^ fabric had a low surface roughness (Ra = 18 um) and a high surface area (2.19 mm^2^ mm^−2^ of fabric area), allowing the ink to penetrate evenly into the fiber bulk due to the strong capillary wicking force.

The patch design requires very high resolution for the transmission lines (~1.2 mm) and the gaps (~1.5 mm), which can be challenging for fabrication on a textile surface. To achieve the high resolution, we used a vinyl mask placed onto the nonwoven fabric and applied uniform pressure and velocity during screen printing to ensure a uniform printed area for the patch design. The printed nonwoven fabric was then cured in an oven at 130 °C for 15 min. The same process was repeated for the ground design (i.e., conductive backing). To form the final antenna assembly, the nonwoven patch design, nonwoven ground design, and dielectric printed knit fabrics were heat laminated together with the TPU webs (Figure 2c).

A separate TPU web was used as an encapsulation layer to protect the antenna. Two standard SubMiniature version A (SMA) connectors with a 50 Ω characteristic impedance were soldered with Chipquik SMDSWLTLFP32 (a low-melting-temperature solder) onto the patch and ground patterns to form a connection. The metal in the solder melted at 170 °C, which enabled robust connections without damaging the textile component of the antenna. An additional UV-curable encapsulation (Dymax 9001-E-V 3.5) was applied to the edges of the solder areas between the textile antenna and connectors. The encapsulation protected the antenna from breaking and ensured a strong mechanical and electrical connection between the textile antenna and SMA connectors. The final product can be seen in Figure 2d.

### 2.5. Characterization of the Antenna

A buckling test was conducted with a commercial tensile tester (Instron, model 5566) to compare the flexibility of different substrates. The sample (8 cm× 8 cm) was gripped between two claps, where the lower clamp was fixed while the upper clamp moved at 50 mm/min. The tested distance between the two clamps was 70 mm.

The performance of the assembled antenna was tested with Agilent E5071C VNA. The antenna was connected to a VNA using two coaxial cables with SMA connectors to measure the reflection coefficient and mutual coupling. The radiation test was conducted in an anechoic chamber, as shown in Supplementary Figure S1. More information about the radiation test can be found in Supplementary Figure S2.

## 3. Results and Discussion

### 3.1. Characterization of BT Dielectric Ink

**Figure 3:**
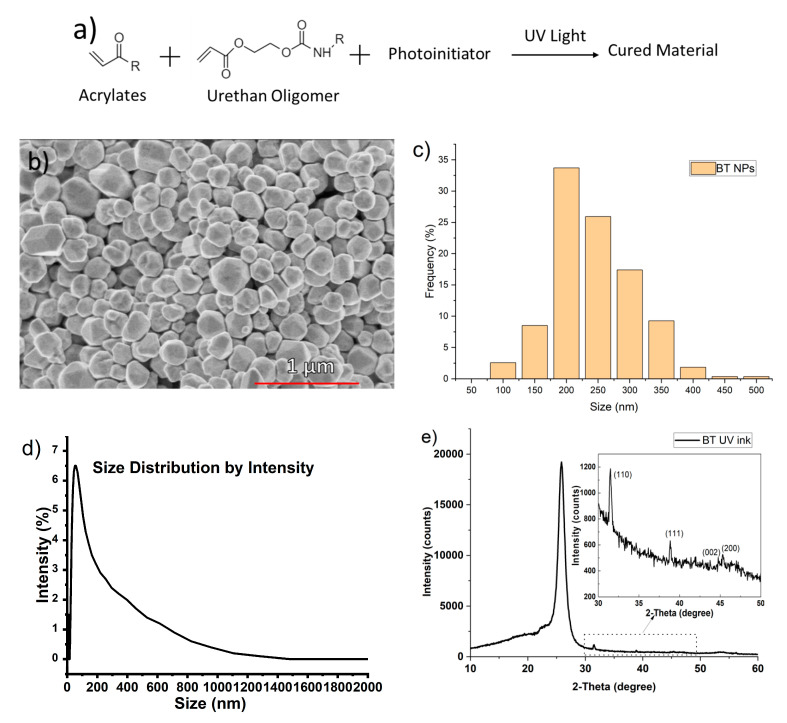
Fundamental characteristics of UV-curable ink with BT nanoparticles. (**a**) Chemical reaction mechanism of acrylate and urethane UV-curing process [43]. (**b**) Scanning electron microscopy (SEM) of BT nanoparticles (50,000×; 1 μm of scale bar). (**c**) Size distribution of the BT nanoparticles. (**d**) BT nanoparticle size distribution according to intensity of UV-curable ink tested by DLS. (**e**) X-ray diffraction (XRD) result of the BT dielectric ink after UV curing.

BT nanoparticles were added to a blend of urethane and acrylates (C3-D1-1032 Dielectric photopolymer, Chem3, LLC, USA) to fabricate the polymer/ceramic ink. Processability at room temperature is essential for use in E-textiles, as textile materials cannot sustain high processing temperatures [39]. The ink with the photoinitiator using ultraviolet radiation can be polymerized after UV curing, and forms a thin film on the textile substrate (Figure 3a). Moreover, the small molecules with low viscosity can help prevent the BT dielectric ink from blocking the spray nozzle (Figure 3a). Figure 3b displays a scanning electron microscopy (SEM) image of the BT nanoparticles, where it was found that the nanoparticles were a mixture of cubic and polyhedric in shape. The size distribution of the BT nanoparticles shown in Figure 3c was obtained by measuring more than 200 particles. The BT nanoparticles used in the dielectric ink exhibited an average size of 211 nm. The size distribution of the BT dielectric ink was tested by a dynamic light scattering (DLS) method. The main peak of the ink is at less than 1000 nm, indicating that the ink dispersed well only with slight aggregation (Figure 3d). This is an indication that the nanoparticle ink will not block the spray nozzle. The viscosity of the BT-UV curing ink may affect the rheological characteristics through printer nozzles, where a high-viscosity ink could lead to nozzle clogging. The BT dielectric ink exhibited Newtonian behavior with a low viscosity of 12 cps, which ensured that the ink could be easily spray coated. The ink surface tension was 37.53 mN/m, and the pendent volume was 3.43 μL. Ink with low surface tension can prevent nozzle dripping and guarantee decent wettability [40,41]. Figure 3e shows the X-ray diffraction (XRD) pattern of BT dielectric ink after UV curing. The BT dielectric ink shows XRD peaks at 25.85° from the photopolymer. The peaks at 31.50° and 38.85° indicate that the BT nanoparticles exhibit a tetragonal crystal structure, which has a higher dielectric performance compared to cubic BT phase [42].

### 3.2. Dielectric Properties of the Printed Textile-Based Antenna

Table 2 provides a list of the dielectric properties of various assembly methods with textile layer combinations and the inclusion of spray-coated BT dielectric ink. In the previous study, the textile antennas were made from eight-layer nonwoven fabrics [44], with a dielectric constant and dielectric loss tested by SPDR of 1.75 and 0.008, respectively. The newly designed textile antenna substrate made of six knit fabrics spray coated with BT dielectric ink sandwiched between two nonwoven fabrics (Figure 2) showed a similar thickness (1.97 mm) and dielectric constant (1.78) to those of the previous study. The direct-write spray-coating process of BT dielectric ink successfully increased the dielectric constant of the six-layered knit fabric structure from 1.43 to 1.61. This demonstrates the potential of using BT dielectric ink to manipulate the dielectric properties of porous materials. As previously mentioned, the dielectric loss can influence the radiation efficiency of the antenna, for which a smaller value is preferred [45]. Although the dielectric loss increased from 0.009 to 0.013 by adding the BT dielectric ink into the knit structure, this is still low compared to the previous studies on textile antennas [25].

### 3.3. Characterization of the Printed Textile-Based Antenna

**Figure 4:**
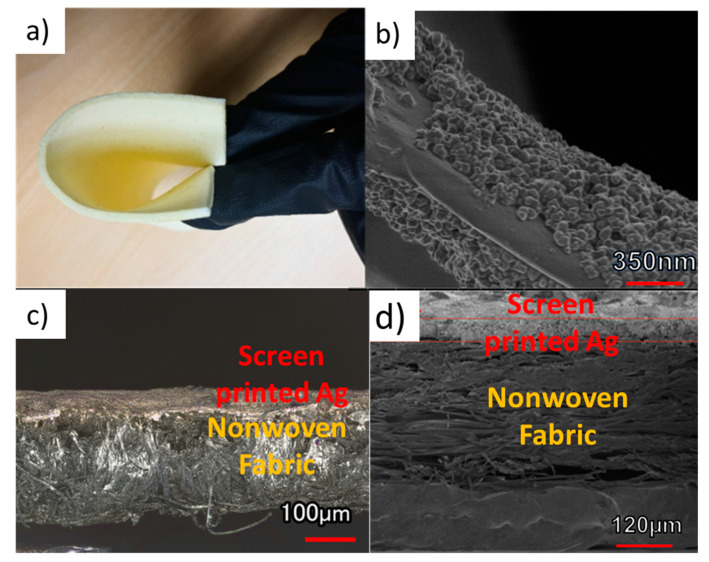
(**a**) Flexibility of the knit fabric assembly with BT dielectric ink and (**b**) an SEM image of the BT with acrylate and urethane UV-curable dielectric ink coated on a single fiber within the fabric after UV curing for 5 min (10,000×, 350 nm of scale bar). (**c**) Cross-sectional microscope image of Ag screen-printed patch section of the antenna (20×, 100 μm of scale bar). (**d**) Cross-sectional SEM image of Ag screen-printed patch section of the antenna (150×, 120 μm of scale bar).

The dielectric-ink-sprayed knit fabric is very flexible, as can be observed in Figure 4a. Figure 4b shows the BT nanoparticles deposited on the textile fiber after spraying. Nonwoven fabric screen printed with silver conductive ink is shown in Figure 4c,d. The silver conductive ink was successfully deposited on the nonwoven fabric and forms a uniform layer around 40 μm thick.

**Figure 5:**
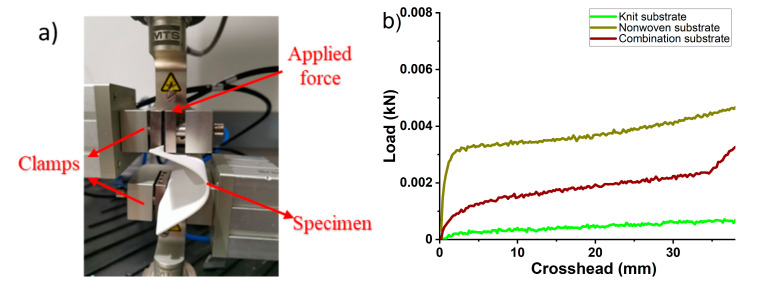
Buckling test of the nonwoven substrate (eight layers of nonwoven fabric), knit substrate (eight layers of knit fabric), and combination substrate (two layers of nonwoven fabric and six layers of knit fabric spray coated with BT dielectric ink). (**a**) Photography of experimental setup. (**b**) Buckling force displacement curve with fixed–fixed ends.

The buckling analysis of the antenna substrate under compression was executed to compare the flexibility of the nonwoven substrate (eight layers of nonwoven fabric), knit substrate (eight layers of knit fabric), and combination substrate (two layers of nonwoven fabric and six layers of knit fabric spray coated with BT dielectric ink). Figure 5a displays the shape of the fabric deflection during the test. The buckling of the fabric under the continuously increasing compression loading force was recorded in Figure 5b. A total of 4 N is needed to cause 30 mm displacement for the nonwoven fabric assembly, while only 0.7 N is necessary for the knit fabric substrate. The larger force required to deform the nonwoven fabric by 30 mm indicates that the nonwoven fabric is more rigid than the knit fabric, since it takes more mechanical energy to displace the nonwoven fabric. Regarding the combination substrate (two layers of nonwoven fabric and six layers of knit fabric spray coated with BT dielectric ink), only 2 N was required. The lower force required for the amalgamated substrate indicates that it behaves like a composite structure and demonstrates superior flexibility compared to the nonwoven substrate.

### 3.4. Simulated and Measured S-Parameter Results for the Printed Textile-Based Antenna

**Figure 6:**
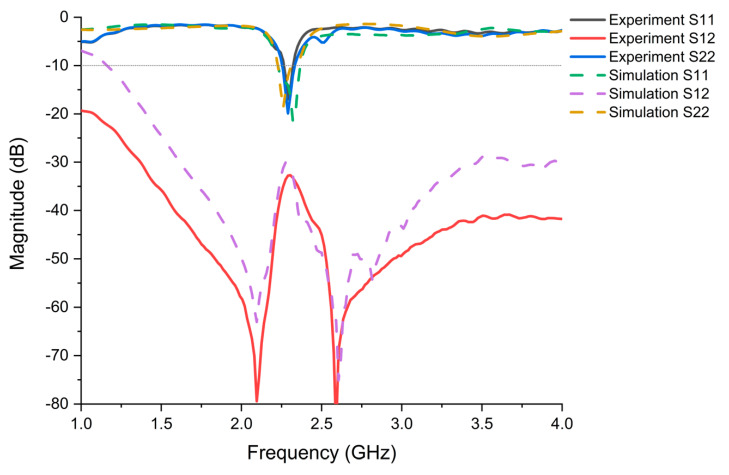
Simulated and measured S-parameters of the proposed full-duplex textile antenna with the combination structure (two layers of nonwoven fabric and six layers of knit fabric spray coated with BT dielectric ink). Experimental data are represented by solid curves, while simulation data are represented by dotted curves.

Full-wave simulations of the antenna were performed using an ANSYS HFSS (Figure 6). A prototype of the complete antenna design was been constructed and tested to verify its impedance matching and mutual coupling performance. SMA connectors were used to feed both antennas. Two-port reflection coefficient measurements of the prototype antenna were performed using a VNA. The simulated and measured S-parameter results of the proposed two-port antenna with optimized dimensions (Figure 1) are provided in Figure 6. The antenna exhibits a −10 dB bandwidth around 79 MHz from 2.26–2.339 GHz, with mutual coupling of less than -30 dB over the entire band. The experimental and simulated results show good agreement for this antenna.

### 3.5. Radiation Pattern and Far-Field Results for the Printed Textile-Based Antenna

**Figure 7:**
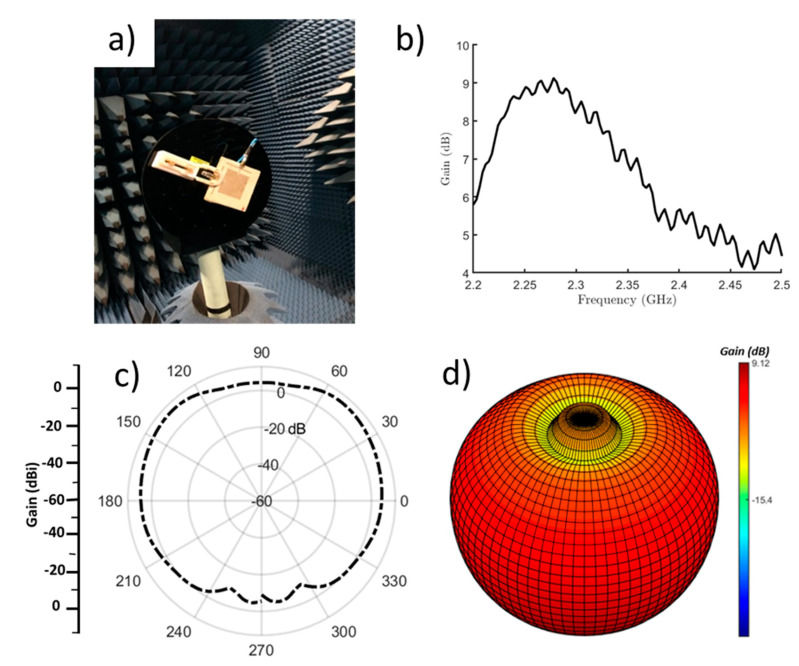
(**a**) Experimental setup for the textile antenna with the combination substrate (two layers of nonwoven fabric and six layers of knit fabric spray coated with BT dielectric ink) for 3D radiation pattern measurements in an anechoic chamber. (**b**) Maximum gain plot at φ = 0°. (**c**) Two-dimensional radiation pattern of the textile antenna at 2.3 GHz and φ = 0°. (**d**) Three-dimensional radiation pattern of the textile antenna plotted in dB at 2.3 GHz.

The radiation pattern of the antenna was measured in an anechoic chamber (Figure 7a). The measurement chamber system was controlled by a computer and equipped with a closed-circuit television camera to visualize the measurements. According to previous work [23], a diagonally directed surface current can be predicted as a combination of both the surface current of the conductive patch and the orthogonally polarized current of the load strips. This yields diagonally polarized far-field radiation, which differs from a conventional patch antenna. The broadside gain profile is provided in Figure 7b. Clearly, a 9.12 dB maximum gain was measured at the on-resonance frequency, around 2.3 GHz. This result is a significant improvement over the gain (5.5 dB) of typical textile patch antennas that have been previously reported [46]. The E-plane radiation pattern at 2.3 GHz for the proposed textile antenna with the amalgamated structure (two layers of nonwoven fabric and six layers of knit fabric spray coated with BT dielectric ink) is provided in Figure 7c, while the corresponding 3D radiation pattern is shown in Figure 7d. These results indicate that the antenna exhibits a uni-directional radiation performance.

### 3.6. Related Work

Table 3 shows the comparison between the proposed antenna and recent work reported in the literature, including antenna material, size, bandwidth, and gain. It can be seen from Table 3 that our proposed antenna has a relatively high gain. Knit fabric is the most widely used fabric in our daily clothing due to its superior flexibility and breathability, but its poor dielectric properties limit its applications in wearable antennas. In this work, the dielectric constant of knit fabric was enhanced by the printing of BT dielectric ink. Instead of using densely woven fabric, filter paper, and PDMS (Polydimethylsiloxane), as shown in Table 3, we employed a combination textile substrate (six layers of knit fabric and two layers of nonwoven fabric) that shows great flexibility and is more suitable for wearable applications. The success of using BT dielectric ink to modify the dielectric properties of porous materials could expand the options of textile materials as antenna substrates.

## 4. Conclusions

In this work, a flexible textile-based full-duplex antenna that employs both knit and nonwoven fabrics was demonstrated. The dielectric constant of the flexible knit fabric assembly was enhanced by a direct-write spray coating of BT nanoparticle dielectric ink, making it suitable for integration into a textile-based antenna. This has demonstrated the potential of using BT dielectric ink and spray coating to manipulate the dielectric properties of porous materials. The resultant flexible antenna is dual-mode (i.e., able to simultaneously operate in transmit and receive modes with very high isolation), is robust to bending, and exhibited a gain of 9.12 dB, which is better than typical textile antennas. The textile antenna in this work shows great promise for use as a multifunctional flexible antenna in E-textile systems.

In future studies, we propose exploring additional knit structures that can further reduce the porosity of this layer as well as more dielectric inks, which can aid in increasing the dielectric constant beyond what is reported in this work. In terms of manufacturing, we also propose the use of alternative direct ink writing technologies, such as inkjet printing, in order to enable more customized design. Finally, the incorporation of these devices into textile panels that can be easily implemented in standard cutting and sewing processes would enable their application.

## Figures and Tables

**Table 1:** Specification of the fabric for antenna design.

Fabrics	Material	Thickness (mm)	Weight (g/m^2^)
Nonwoven Fabric	70 wt.% Polyester/30 wt.% Polyamide	0.33	95
Knit Fabric	Polyester	0.31	145

**Table 2:** Fabric dielectric properties.

Fabric Assembly	Dielectric Constant	Dielectric Loss	Thickness (mm)
Knit Fabric, six Layers	1.43	0.009	1.68
Knit Fabric, six Layers w/BT Dielectric Ink	1.61	0.013	1.60
Knit Fabric, six Layer w/BT Dielectric Ink + two Layers Nonwoven Fabric	1.78	0.021	1.97
Nonwoven Fabric, eight Layers	1.75	0.008	1.94

**Table 3:** Comparison of the antennas proposed in previous works.

Reference	Material	Substrate Dielectric Constant and Loss	Volume (mm^3^)	Bandwidth (MHz)	Gain (dBi)
[47]	Screen-printed Ag ink and PDMS	-	60 × 60 × 2	105	6.987
[48]	Inkjet-printed Ag layer and 65/35 polyester cotton woven fabric	1.229 and 0.001	37.4 × 28.1 × 1.6	24.5	5.09
[49]	GO film and cellulose filter paper	3.06, -	119.4 × 70 × 0.46	100	12
[11]	Copper sheet and jean fabric	1.7 and 0.025	40 × 20 × 0.3	300	4.48
[50]	Copper sheet and jean fabric	1.7 and 0.085	100 × 100 × 6	240	2.42
[51]	Nickel-/copper-plated textile and thick felt textile material	1.2 and 0.044	150 × 150 × 4	120	3.5
Proposed	Screen-printed Ag layer and nonwoven/knit fabric spray coated with BT dielectric ink	1.78 and 0.012	80 × 80 × 2	79	9.12

## Supplementary Materials

The following are available online at https://www.mdpi.com/2072-666X/11/12/1056/s1: Figure S1: Images of radiation measurements in an anechoic chamber: (a) front view and (b) side view, Figure S2: Schematic diagram of the experimental setup for the antenna radiation test.
