# Supplementary material for: Direct-Write Spray Coating of a Full-Duplex Antenna for E-Textile Applications
Source: Micromachines (Basel). 2020 Nov 29;11(12):1056. doi: 10.3390/mi11121056 (PMC7760154; doi:10.3390/mi11121056)
Supplement: Supplementary file 1 [file micromachines-11-01056-s001.pdf]

micromachines

Supplementary Information: Direct-Write Spray Coating of a Full-Duplex Antenna for E-Textile Applications

Ying Zhou [1], Saber Soltani [2], Braden M. Li [1], Yuhao Wu [2], Inhwan Kim [1], Henry Soewardiman [3], Douglas H. Werner [2] and Jesse S. Jur [1,*]

[1] Department of Textile Engineering, Chemistry and Science, North Carolina State University, Raleigh, NC 27606, USA; yzhou32@ncsu.edu (Y.Z.); bmli@ncsu.edu (B.M.L.); ikim7@ncsu.edu (I.K.)
[2] Electrical Engineering Department, The Pennsylvania State University, University Park, PA 16802, USA; saber_soltani@psu.edu (S.S.); yuw476@psu.edu (Y.W.); dhw@psu.edu (D.H.W.)
[3] Mechanical and Aerospace Engineering, North Carolina State University, Raleigh, NC 27606, USA; hsoewar@ncsu.edu
* Correspondence: jsjur@ncsu.edu

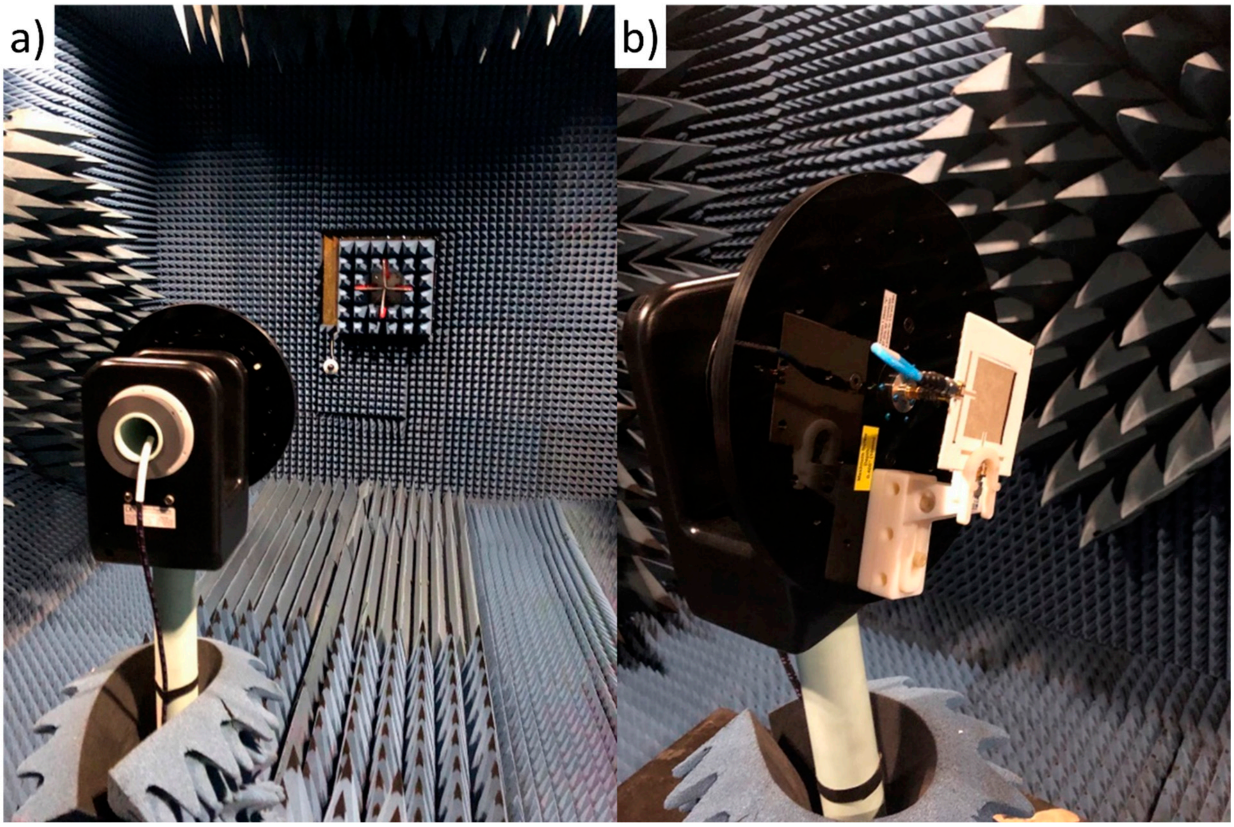

Figure S1. Images of radiation measurements in an anechoic chamber: (**a**) front view and (**b**) side view.

Figure S2 shows the schematic diagram of the setup for the antenna radiation test. A Diamond engineering D6050 positioner was used to take measurements, controlled using a Desktop Antenna Measurement System (DAMS) software. An ETS_Lindgren_3164-10 Horn antenna was employed as a reference antenna with known characteristics (gain, pattern, etc.). The distance between the transmitter and receiver antennas inside the anechoic chamber was set to 3.72 m. A four-port Keysight Agilent N5227B Performance Network Analyzer (PNA) was used for injecting energy into the antenna under test (AUT) with port 1 as an RF power transmitter and ports 2 and 4 as a receiver system. The D6050 positioner was also used to rotate the test antenna relative to the source antenna so as to measure the radiation pattern as a function of the angle.

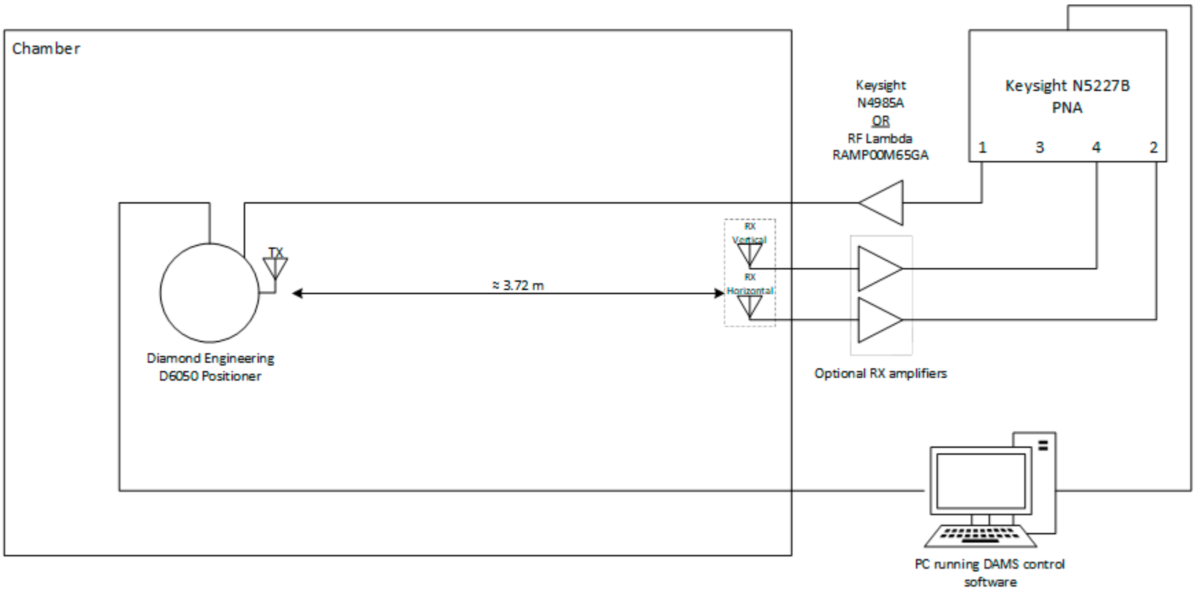

Figure S2. Schematic diagram of the experimental setup for the antenna radiation test.
